# Antifungal Activity Assessment of Postbiotic Mediators Derived From *Lactobacillus rhamnosus* GG and *Lactobacillus reuteri* Against *Candida albicans*

**DOI:** 10.1002/mbo3.70439

**Published:** 2026-10-11

**Authors:** Fatemeh Amirinia, Morteza Banakar, Mojgan Alaeddini, Shahroo Etemad‐Moghadam, Maryam Pourhajibagher, Maryam Noori

**Affiliations:** ^1^ Dental Research Center, Dental Research Institute Tehran University of Medical Sciences Tehran Iran; ^2^ Department of Medicine and Mycology, School of Medicine Shiraz University of Medical Sciences Shiraz Iran; ^3^ Department of Medical Biotechnology Shahid Sadoughi University of Medical Sciences Yazd Iran; ^4^ Department of Research Analytics, Saveetha Dental College and Hospitals, Saveetha Institute of Medical and Technical Sciences Saveetha University Chennai India; ^5^ Food and Water Disease Research Center, Research Institute for Gastric and Liver Disease Shahid Beheshti University of Medical Sciences Tehran Iran

**Keywords:** antifungal effects, biofilm inhibition, *Candida albicans*, *Lactobacillus*, oral candidiasis, postbiotics

## Abstract

The rise of antimicrobial resistance has increased interest in safe alternative therapeutic agents, including postbiotic mediators (PMs), which are sterile, cell‐free, filter‐sterilized preparations derived from probiotics. This study investigated the antifungal and antibiofilm activity of PMs derived from *Lactobacillus rhamnosus* GG (LGG) and *Lactobacillus reuteri* (LR) against *Candida albicans*, an opportunistic pathogen implicated in oral infections. PMs were prepared from LGG and LR cultures. The minimum inhibitory concentration (MIC) and minimum fungicidal concentration (MFC) against *C. albicans* were determined using broth microdilution and colony‐forming unit (CFU) counts. Antibiofilm activity at sub‐MIC concentrations was evaluated by assessing metabolic activity (XTT assay) and biofilm architecture (scanning electron microscopy, SEM). The MIC and MFC of PMs from both LGG and LR were 25% (v/v). Both PMs significantly reduced the metabolic activity of *C. albicans* biofilms as measured by the XTT assay (*p* ≤ 0.002); however, a statistically significant reduction in viable biofilm counts was observed only with LGG PMs (*p* = 0.0038), whereas LR PMs showed a non‐significant reduction (*p* = 0.1537). SEM showed marked changes in biofilm architecture. PMs from LGG and LR showed in vitro antifungal activity against *C. albicans*, with both preparations suppressing biofilm metabolic activity and LGG PMs additionally producing a statistically significant reduction in viable biofilm counts. These findings support further investigation of *Lactobacillus*‐derived PMs as candidate adjunctive antifungal approaches; however, validation in clinical and antifungal‐resistant isolates, chemical and mechanistic characterization, cytotoxicity testing, and in vivo studies are required before clinical application.

## Graphical abstract

Postbiotic metabolites derived from *Lactobacillus rhamnosus* GG and *Lactobacillus reuteri* demonstrate potent antifungal and anti‐biofilm activity against *Candida albicans*. By significantly impairing fungal viability, metabolic activity, and biofilm structural integrity, these non‐viable compounds offer a promising, safe alternative for managing oral candidiasis and preventing dental caries.

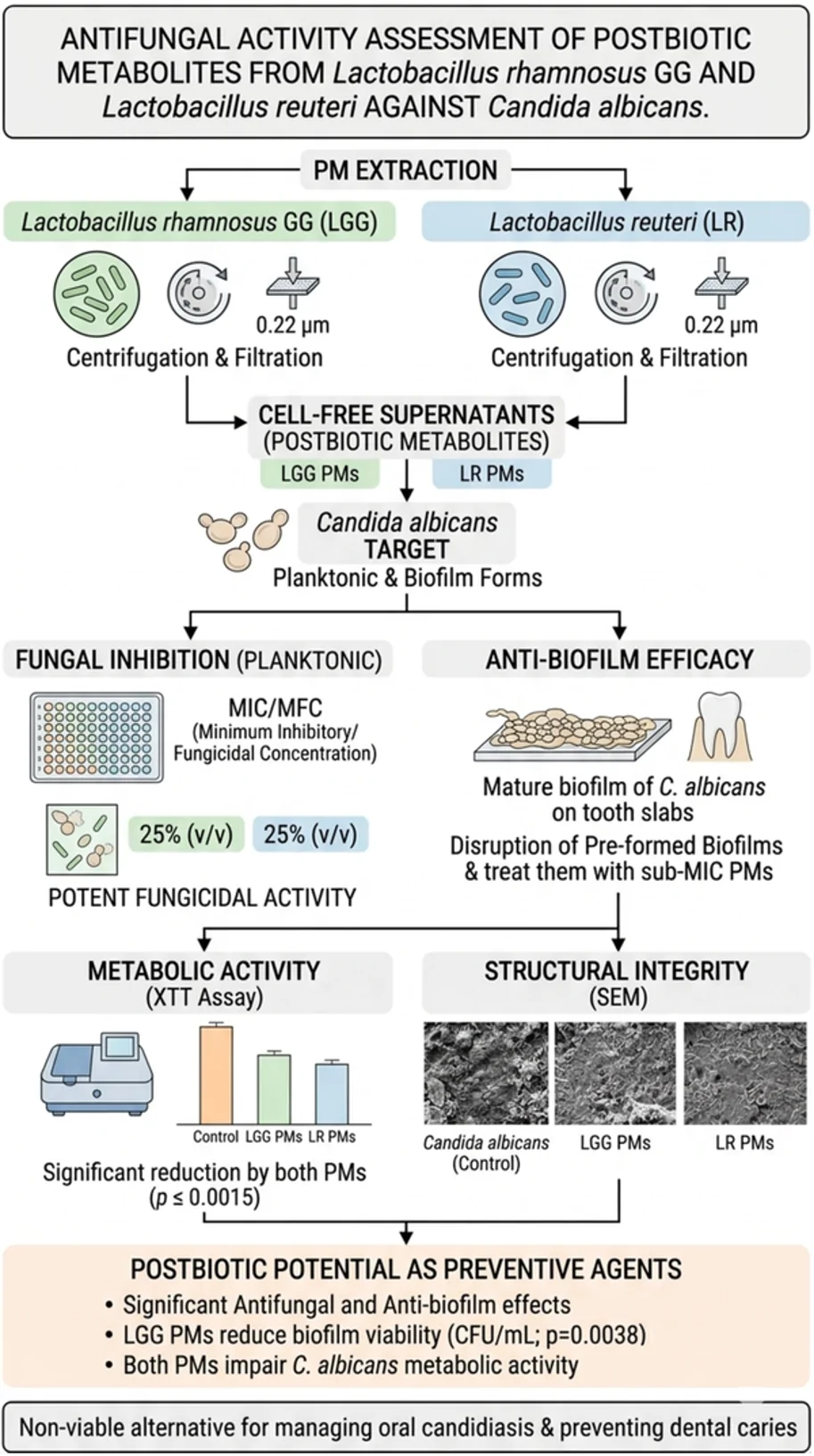

## 1 Introduction

*Candida albicans* (*C. albicans*) is a naturally occurring oral yeast that can contribute to dental caries and oral infection (Cao et al. 2025; Ev et al. 2020; Li et al. 2014). This yeast colonizes various surfaces, including the tongue and dental plaque (Rawnuck et al. 2025). However, under conditions that disrupt the oral microbial balance, it can proliferate excessively and lead to infection (Cao et al. 2025). *C. albicans* can be present in decayed teeth, particularly in dentin, and may contribute to tissue degradation by secreting collagenolytic enzymes (Lu et al. 2023). These enzymes can break down dentinal collagen, increasing permeability and causing damage to the dental matrix (Du et al. 2021). *C. albicans* can also promote demineralization of hard dental tissues by lowering the pH of its environment (Eidt et al. 2019). Therefore, this yeast may contribute to the progression of dental caries in conjunction with other microorganisms (Regis et al. 2025).

The overuse and misuse of antifungal agents have contributed to the emergence of resistant *C. albicans* strains, posing significant therapeutic challenges (Abu‐Humaidan et al. 2025; Mudenda 2024). Studies have reported resistance of *C. albicans* to drugs such as fluconazole and itraconazole, which can contribute to chronic and recurrent infections (Mudenda 2024; Rawnuck et al. 2025; Seyedjavadi et al. 2024). In addition, non‐albicans *Candida* species are increasingly recognized as treatment challenges (Raposa and Vazquez 2025; Rawnuck et al. 2025). Given the growing resistance of *C. albicans* to conventional antifungals, alternative therapeutic approaches, including postbiotic metabolites, warrant investigation. However, studies evaluating the direct effects of PMs on fungal biofilms remain limited.

As live microorganisms, probiotics may play a role in managing oral candidiasis (Rajão et al. 2025). They can suppress the growth and colonization of *C. albicans* and help maintain oral microbial balance by producing antifungal compounds such as lactic acid and bacteriocins (Homayouni Rad et al. 2023; Pallavi et al. 2024; Rajão et al. 2025; Sharifzadeh et al. 2023). Potential benefits of probiotics include reduced adverse effects associated with antifungal medications and immunomodulatory effects (Wu et al. 2022). However, potential drawbacks include delayed effects, variable efficacy among individuals, and an increased risk of infection in immunocompromised patients (Wu et al. 2022).

Unlike probiotics, which are live microorganisms, postbiotics comprise non‐viable microbial preparations or their components that retain bioactive properties (Prajapati et al. 2023). Postbiotic mediators (PMs), defined herein as sterile, cell‐free, filter‐sterilized preparations derived from probiotic bacterial cultures and containing bioactive metabolites, are products of probiotic metabolic activity (Hill et al. 2021; Chakraborty et al. 2025; Samuel et al. 2024; Yeşilyurt et al. 2021). Compared with live probiotics, cell‐free PM preparations avoid administration of viable microorganisms and may offer practical advantages in storage and formulation; nevertheless, the safety and stability of each preparation require direct product‐specific evaluation (Ji et al. 2023; Prajapati et al. 2023). These characteristics make PMs of particular interest for populations in whom administration of live microorganisms may be undesirable, while formal biocompatibility assessment remains necessary (Fang et al. 2025; Ji et al. 2023).

Different *Lactobacillus* strains have long been recognized as effective probiotics (Chakraborty et al. 2025); however, the number of studies on their PMs remains limited. *Lactobacillus rhamnosus* GG (LGG) and *Lactobacillus reuteri* (LR) are well‐studied probiotic strains with reported antimicrobial activity. They are known for robust growth, adhesion, pathogen antagonism, and production of antimicrobial substances, and are therefore considered promising probiotic strains. The strain‐specific effects of various Lactobacillus isolates, their metabolites, and the mechanisms underlying activity against *C. albicans* remain unclear. Therefore, this study aimed to investigate the antifungal effects of PMs derived from LGG and LR by assessing their antibiofilm activity and effects on the metabolic activity of *C. albicans*.

## 2 Methods

### 2.1 Culture Strains and Growth Conditions

*L. rhamnosus* GG (ATCC 53103) and *L. reuteri* (ATCC 23272) were selected because of their well‐documented probiotic properties and prior reports of antimicrobial activity against oral pathogens (Banakar et al. 2023). They were maintained on MRS agar (de Man, Rogosa, and Sharpe agar) (Merck, Darmstadt, Germany), subsequently cultured in MRS broth (Merck, Darmstadt, Germany), and incubated in a CO_2_ incubator at 37°C ± 1°C for 48 h. *C. albicans* ATCC 10231 was obtained from the Iranian Biological Resource Centre (Tehran, Iran) and incubated on Sabouraud dextrose agar (SDA, Merck, Darmstadt, Germany) at 35°C ± 1°C for 24 h.

Five colonies with a diameter of approximately 1 mm were selected from a 24‐h culture. These colonies were suspended in 5 mL of sterile saline and vortexed for 15 s. The yeast cell density was standardized to 0.5 McFarland turbidity (1–5 × 10^6^ CFU/mL) using sterile saline, as measured spectrophotometrically at 600 nm. The suspension was then diluted with RPMI 1640 medium (with glutamine and without bicarbonate) (QLab, Canada) buffered with 0.165 mol/L MOPS (3‐(N‐morpholino)propanesulfonic acid) to achieve a final concentration of 5.0 × 10^2^ to 2.5 × 10^3^ yeast cells/mL for MIC/MFC susceptibility testing, in accordance with CLSI M27‐A3 guidelines (Banakar et al. 2023; Cilo and Ener 2021; El‐Mashad et al. 2012; Rodríguez‐Tudela et al. 2003). For biofilm formation assays (biofilm destruction and XTT reduction assay), a higher inoculum of 1.5 × 10^6^ CFU/mL was used, consistent with established protocols for in vitro *C. albicans* biofilm generation, which require a higher initial cell density to ensure confluent and reproducible biofilm formation within the incubation period (Matsubara et al. 2016; Rossoni et al. 2018).

### 2.2 Postbiotic Mediators

After 48 h of incubation, the tubes containing the culture medium and *Lactobacillus* species were centrifuged at 10,000*g* for 10 min at 4°C. The supernatant was then separated from the sediment and transferred to a new tube. All supernatants were filtered through a 0.22 μm polyethersulfone membrane syringe filter (Millipore, Burlington, MA, USA). The pH of the supernatants was adjusted to physiological pH (7.2–7.4) using 5 M sodium hydroxide before use (Banakar et al. 2023). This pH adjustment may alter the antimicrobial activity of organic acid constituents (e.g., lactic acid and acetic acid), as these compounds exert their primary antifungal effects in their protonated, acidic form; this consideration is discussed further in the Limitations section.

### 2.3 Evaluation of Antifungal and Antibiofilm Activity

#### 2.3.1 Determination of Minimum Inhibitory Concentration and Minimum Fungicidal Concentration of Postbiotic Mediators

The minimum inhibitory concentration (MIC) and minimum fungicidal concentration (MFC) were determined according to the Clinical and Laboratory Standards Institute (CLSI) M27‐A3 broth microdilution guidelines (Clinical and Laboratory Standards Institute 2008, 2017). Briefly, 100 μL of RPMI 1640 medium buffered with 0.165 mol/L MOPS was dispensed into wells 2–11 of each row in round‐bottom 96‐well microplates. One hundred microliters of undiluted PM (100% v/v) was added to well 1, and serial twofold dilutions were prepared by transferring 100 μL aliquots sequentially from well 1 through well 10, with thorough mixing at each step; the final 100 μL from well 10 was discarded. One hundred microliters of the standardized *C. albicans* inoculum (5.0 × 10^2^ to 2.5 × 10^3^ cells/mL in RPMI‐MOPS) was then added to all wells, resulting in a final assay volume of 200 μL and nominal PM concentrations ranging from 50% (v/v) downward. Plates were incubated at 37°C for 24 h. The MIC was recorded as the lowest PM concentration showing no visible turbidity. Appropriate positive (*C. albicans* in medium alone) and negative (PMs in medium alone) controls were included. All determinations were performed in triplicate.

Subcultures from the broth dilution wells were used to determine the MFC, which was defined as the lowest concentration of PMs that prevented the growth of *C. albicans* on SDA. MFC was also defined as the lowest concentration of an antifungal agent that reduced the initial inoculum by at least 99.99%. All experiments were repeated at least three times.

#### 2.3.2 Determination of the Biofilm Destruction Effect of PMs

A suspension of *C. albicans* with a concentration of 1.5 × 10^6^ CFU/mL was prepared, and 200 μL was added to each well of a flat‐bottomed 96‐well microplate. The microplate was incubated at 37°C for 24 h to form a *C. albicans* biofilm. After the incubation period, the suspension was aspirated, and the wells were washed three times with sterile PBS at pH 7.4. Subsequently, 100 μL of each PM at a concentration of 2 × MIC was added to each well, and the microplate was incubated at 37°C for 24 h. After incubation, the *C. albicans* biofilm was scraped from the bottom of the microplate and serially diluted for plating to determine CFU/mL. All experiments were performed in triplicate (Esfandiary et al. 2024).

### 2.4 Microscopic Visualization of Biofilm

According to a previous study, tooth slabs were prepared for microbial biofilm formation (Banakar et al. 2023). *C. albicans* biofilms were formed on the tooth slabs by incubating the slabs in a *C. albicans* suspension at 1.5 × 10^6^ CFU/mL for 48 h at 37°C. The slabs were then washed three times with sterile PBS and treated with each PM at a concentration of 2 × MIC for 24 h at 37°C (Banakar et al. 2023). After completing the incubation period, the biofilm on the teeth was fixed with 2.5% glutaraldehyde. The samples were subsequently dehydrated using ascending concentrations of ethanol (50%, 60%, 70%, 80%, 90%, and two rounds of 100%) and coated with a thin layer of palladium‐gold for visualization (Banakar et al. 2023; Jeyarajan et al. 2025).

### 2.5 Evaluation of Metabolic Activity Using the XTT Reduction Assay

To measure the metabolic activity of *C. albicans* biofilms and avoid effects related to cell death caused by the MIC, biofilms were assessed using the XTT reduction assay kit (2,3‐bis(2‐methoxy‐4‐nitro‐5‐sulfophenyl)‐5‐[(phenylamino)carbonyl]‐2H‐tetrazolium hydroxide) (XTT assay kit; Applied Science Roche, Indianapolis, IN, USA) according to the manufacturer's instructions. Initially, 100 μL of a *C. albicans* suspension at a concentration of 1.5 × 10^6^ CFU/mL was added to a flat‐bottomed 96‐well microplate and incubated at 37°C for 24 h. The cells were then treated with 1/2 × MIC of each PM for 24 h at 37°C. This concentration was selected to study the effects of PMs on metabolic activity while avoiding effects related to cell death caused by the MIC and higher concentrations. After incubation, 50 μL of the XTT solution was added to each well, and the microplate was incubated in the dark at 37°C for 2 h. Absorbance was measured at 490 nm using a microplate reader (Cosio et al. 2025).

### 2.6 Statistical Analysis

Each experiment was performed in triplicate (*n* = 3), and results from the experimental groups are expressed as percentages relative to the control group. Although normality was assessed using the Shapiro‐Wilk test prior to parametric analyses, it is acknowledged that this test has limited statistical power at *n* = 3; results should therefore be interpreted with appropriate caution. Pairwise comparisons of CFU/mL values between each PM treatment group (LGG or LR) and the untreated control were performed using a two‐tailed Student's *t*‐test. Comparisons of metabolic activity (XTT absorbance values) across all three groups (LGG PMs, LR PMs, and control) were performed using one‐way analysis of variance (ANOVA) followed by Dunnett's post hoc test. All results are presented as mean ± standard deviation, and a *p*‐value of less than 0.05 was considered statistically significant.

## 3 Results

### 3.1 MIC and MFC of the Postbiotic Mediators

To determine the lowest concentration of PMs that inhibited the growth of *C. albicans*, serial dilutions of PMs were prepared and tested. The MIC for both LGG‐ and LR‐derived PMs was 25% (v/v), as no visible turbidity was observed at higher concentrations. No growth of *C. albicans* was observed on SDA at concentrations above 25% (v/v), confirming the MFC value. The MFC values were equal to the MIC (25% v/v) for both PMs, indicating fungicidal activity.

### 3.2 Biofilm Destruction of *C. albicans* by Postbiotic Mediators

Colony‐forming unit (CFU/mL) enumeration was used to evaluate the biofilm‐disruption effect of the PMs on *C. albicans* biofilm (Figure 1). The mean CFU/mL values were (3.2 ± 0.25) × 10^7^, (2.64 ± 0.19) × 10^7^, and (3.01 ± 0.28) × 10^7^ for the control, LGG PM, and LR PM groups, respectively. Both PMs yielded lower CFU counts than the control; however, the reduction was statistically significant only in the LGG group (*p* = 0.0038) and not in the LR group (*p* = 0.1537).

**Figure 1:**
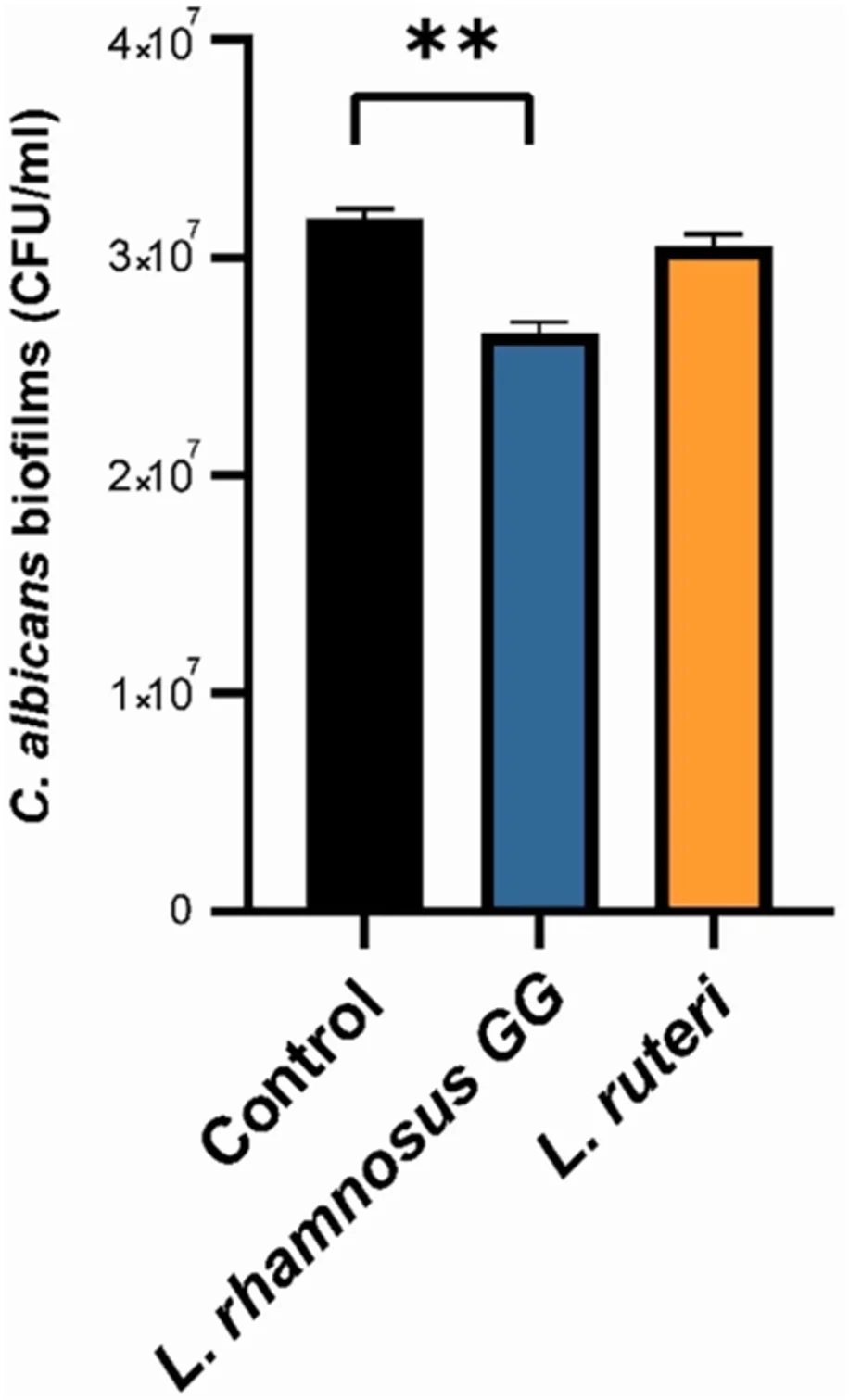
Changes in *Candida albicans* colony‐forming units (CFU/mL) after treatment with postbiotic mediators. **p < 0.01.

### 3.3 Scanning Electron Microscope Analysis

Scanning electron microscopy (SEM) analysis revealed reduced microbial density and fewer microcolonies on the surface following treatment with PMs, indicating changes in biofilm architecture (Figure 2). Consistent with the CFU/mL measurements, LGG‐derived PMs exhibited a more pronounced antibiofilm effect than LR‐derived PMs.

**Figure 2:**
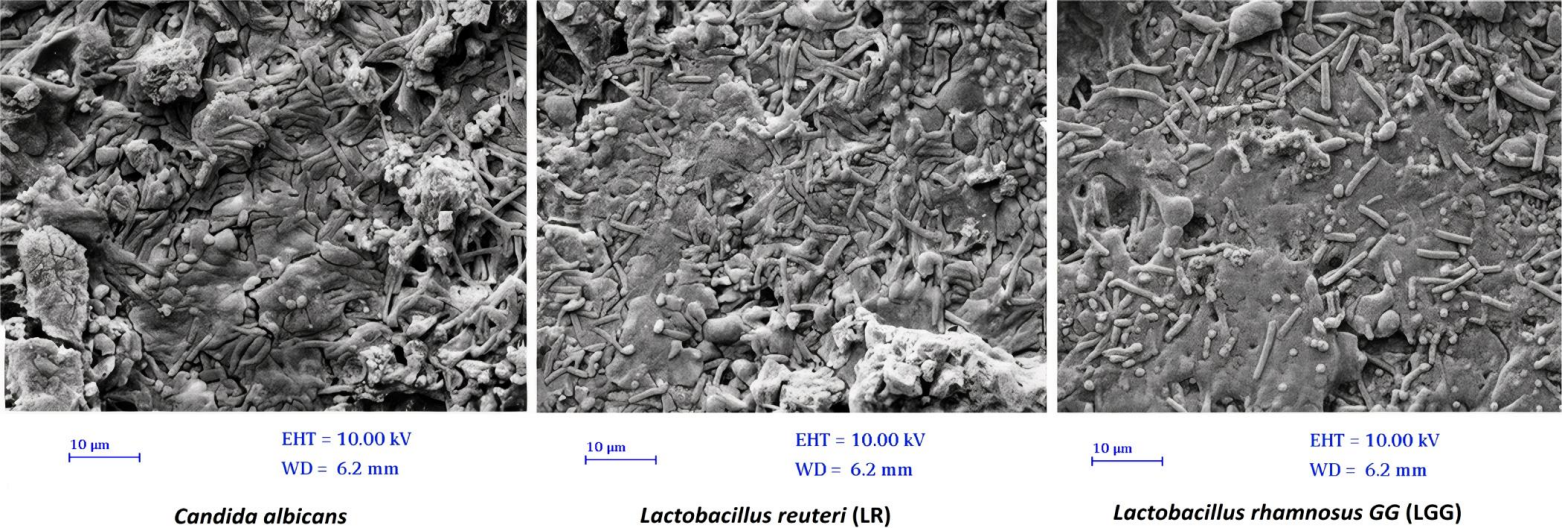
Scanning electron microscopy images of *Candida albicans* biofilms treated with postbiotic mediators (Scale bar = 10 μm; Mag = 1.00 KX).

### 3.4 Inhibitory Effect of PMs on *C. albicans* Metabolic Activity

To measure the metabolic activity of *C. albicans* biofilms, we used the XTT reduction assay (Figure 3). The mean (± SD) absorbance values of the control, LGG PMs, and LR PMs were 1.78 ± 0.09, 0.96 ± 0.06, and 1.12 ± 0.08, respectively. The PMs from both the LGG and LR groups significantly reduced the metabolic activity of *C. albicans* compared with the control (*p* = 0.0009 and *p* = 0.0015, respectively). These findings indicate that PMs reduced *C. albicans* biofilm metabolic activity under the tested conditions.

**Figure 3:**
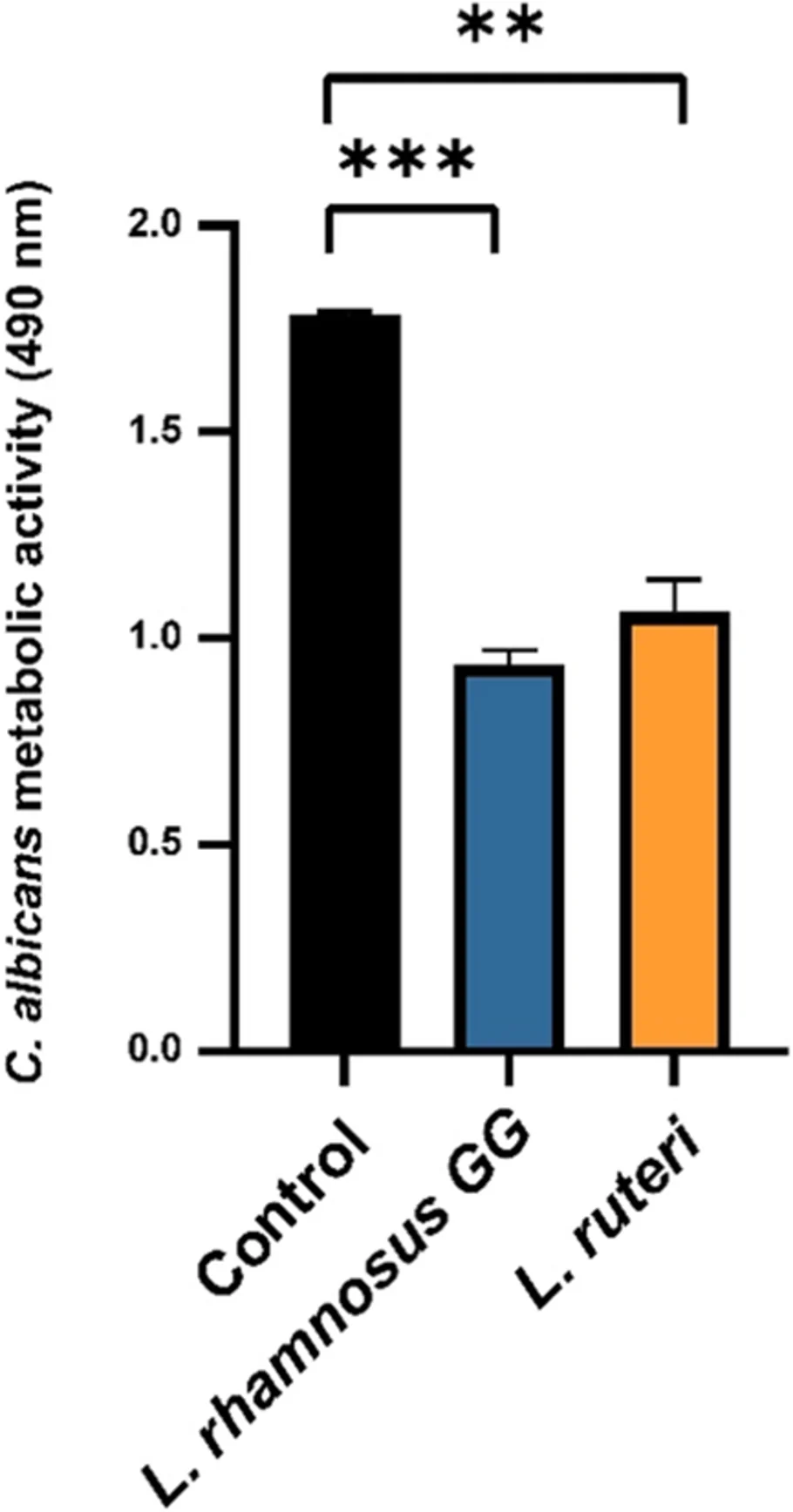
Mean absorbance values of *Candida albicans* metabolic activity based on the XTT reduction assay. *p < 0.01, **p < 0.001. Error bars represent standard deviations.

## 4 Discussion

Probiotic *Lactobacillus* species have received substantial attention for their antimicrobial properties (Ji et al. 2023; Nader‐Macías and De Gregorio 2022; Prajapati et al. 2023), and antibacterial and antifungal effects of these bacteria have been reported (Ji et al. 2023; Matsubara et al. 2016; Prajapati et al. 2023). Among the proposed antifungal mechanisms of *Lactobacillus* species is inhibition of morphogenesis, including suppression of hyphal growth in *Candida spp*. (Abdel‐Nasser et al. 2023). *Lactobacillus* species may also weaken the fungal cell wall through the production of hydrolytic enzymes, such as chitinase and β‐glucanase, potentially increasing susceptibility to other antifungal agents (Abdel‐Nasser et al. 2023). In addition, metabolites produced by *Lactobacillus* species may influence host immune responses and inhibit fungal growth (Abdel‐Nasser et al. 2023; Nader‐Macías and De Gregorio 2022).

PMs comprise bioactive compounds and metabolites produced by probiotic bacteria through metabolic activity or cellular breakdown and have been reported to possess diverse biological properties (Chakraborty et al. 2025; Fang et al. 2025). PMs can contain substances such as bacteriocins, secreted peptides, antifungal proteins, and hydrogen peroxide that may contribute to antifungal activity (Abdel‐Nasser et al. 2023; Chakraborty et al. 2025; Prajapati et al. 2023). Some *Lactobacillus*‐derived metabolites can damage fungal cell membranes, leading to leakage of cell contents and cell death (Abdel‐Nasser et al. 2023; Chakraborty et al. 2025). For example, hydrogen peroxide can cause oxidative stress in fungal cells. In a study, the ability of LGG to produce and secrete the major secretory peptidoglycan hydrolase Msp1 (major secretory protein 1) was shown to contribute to chitin degradation in *C. albicans* hyphae (Allonsius et al. 2019). In addition, lactic acid in LGG‐derived preparations affected the glucan coating on the surface of *C. albicans* hyphae (Ballou et al. 2016). This may make hyphae more accessible to Msp1 activity, thereby contributing to *Candida albicans* damage (Allonsius et al. 2019; Ferreira et al. 2024; Song and Lee 2017). LR can also produce various organic acids, including acetic, lactic, oxaloacetic, propanoic, (hexa‐ and octa‐)decanoic, and benzoic acids, which have been detected in LR PMs and may contribute to growth restriction or fungal cell deformation (Bakhshi et al. 2021).

Postbiotics have been investigated for potential oral‐health applications, including inhibition of microorganisms associated with oral disease (Harat and Pourjafar 2025; Heidari et al. 2025). As noted above, postbiotic compounds may inhibit the growth of fungi and bacteria, including *C. albicans* (Banakar et al. 2023; Sharifzadeh et al. 2023; Wu et al. 2022). Recent studies have suggested that postbiotics may help maintain microbial balance by modulating the oral microbiota and enhancing antifungal activity, potentially reducing the risk of oral infections and dental caries (Harat and Pourjafar 2025; Heidari et al. 2025; Marunganathan et al. 2025). Various studies have also documented antifungal compounds produced by lactobacilli (Bakhshi et al. 2021; Banakar et al. 2023; Marunganathan et al. 2025; Song and Lee 2017).

The increasing prevalence of dental caries and its negative effects on public health and quality of life, especially among children, highlight the need for novel prevention methods (Ehizogie Paul Adeghe 2024). To address this need, we evaluated the antifungal activity of postbiotic mediators from LGG and LR against *C. albicans*. The MIC and MFC results showed that PMs derived from both lactobacilli inhibited *C. albicans*, corroborating previous findings on lactobacilli‐derived compounds (De Gregorio et al. 2019; Ferreira et al. 2024; Rajão et al. 2025). Biofilm maturation is associated with increased resistance to stress and antifungal therapies (Rajão et al. 2025; Zhang et al. 2024). Furthermore, substantial genetic and phenotypic differences exist between biofilm cells and their planktonic counterparts, contributing to increased tolerance and resistance to antimicrobial drugs (Esfandiary et al. 2024; Matsubara et al. 2016; Rajão et al. 2025). Consequently, treatments may be more effective against less organized cellular structures, such as planktonic cells, than against complex biofilms. Therefore, developing antifungal agents capable of targeting planktonic forms and preventing biofilm formation may improve treatment outcomes (Lu et al. 2023; Rajão et al. 2025).

Based on the biofilm assay, the CFU counts for both types of postbiotic mediators were lower than those of the control group. LGG‐derived PMs produced a greater reduction in *C. albicans* viability (CFU/mL) than LR‐derived PMs. It is worth noting that the absolute reduction in viable counts, while statistically significant for LGG PMs, was relatively modest (approximately a 17.5% decrease relative to control, or ~0.08 log_10_). Nevertheless, this viability reduction occurred alongside substantial suppression of metabolic activity (see XTT results below), indicating that the PMs exerted biologically relevant effects on biofilm physiology even though they did not achieve rapid, high‐level killing of biofilm‐embedded cells within the 24‐h exposure window. SEM analysis also revealed marked changes in *C. albicans* biofilm architecture following treatment with PMs derived from both lactobacilli strains. These findings align with previous studies demonstrating inhibitory effects of lactobacilli on *C. albicans* (Bakhshi et al. 2021; El‐Ashmony et al. (2023); Sharifzadeh et al. 2023; Song and Lee 2017).

The observed disparity in biofilm reduction between LGG PMs and LR‐derived PMs, despite comparable MIC/MFC values, may reflect strain‐specific differences in metabolite composition and biofilm‐targeted mechanisms (MacAlpine et al. 2021; Poon and Hui 2023). While MIC/MFC assays primarily assess fungicidal activity against planktonic cells, LGG PMs may contain metabolites—such as biosurfactants, proteases, or bacteriocins—that could destabilize biofilm architecture by affecting extracellular matrices or hyphal formation, a critical virulence trait of *C. albicans* (Jang et al. 2019; Parolin et al. 2015). Furthermore, LGG‐derived metabolites may exhibit enhanced penetration into biofilms or alter metabolic pathways involved in biofilm maintenance (Rossoni et al. 2018). Strain‐specific organic acids (e.g., lactic or acetic acid) could also interact with other antifungal components to impair biofilm viability. These possibilities are consistent with prior studies highlighting *Lactobacillus* strain variability in antimicrobial metabolite profiles and underscore the need for metabolite characterization to optimize postbiotic formulations for biofilm‐related infections (Heidari et al. 2025; Sharifzadeh et al. 2023; Yocheva et al. 2024).

XTT assay results demonstrated that PMs derived from LGG and LR significantly reduced the metabolic activity of *C. albicans* biofilms under the tested conditions. This finding indicates suppression of biofilm metabolic activity, but the XTT assay alone does not establish the molecular mechanism of action or demonstrate that a specific virulence pathway was inhibited. In particular, hyphal transition, fungal membrane integrity, extracellular‐matrix production, and biofilm‐associated gene expression were not directly measured in the present study. Previous investigations have shown that *Lactobacillus*‐derived factors can affect *Candida* filamentation and biofilm‐related phenotypes (Allonsius et al. 2019; Jang et al. 2019; MacAlpine et al. 2021; Matsubara et al. 2016), but whether the LGG and LR PMs tested here act through the same pathways remains to be determined. Accordingly, the present data should be interpreted as evidence of in vitro antifungal and antibiofilm activity that warrants targeted mechanistic confirmation.

Several limitations should be considered when interpreting these findings. First, only one reference strain of *C. albicans* (ATCC 10231) was evaluated; therefore, the results cannot be assumed to represent the susceptibility of genetically diverse clinical isolates, antifungal‐resistant strains, or non‐albicans *Candida* species. Future studies should test a broader panel of clinical isolates, including azole‐resistant strains. Second, the PMs were evaluated as crude cell‐free preparations and were not chemically characterized. Consequently, the concentrations and relative contributions of organic acids, peptides, proteins, bacteriocin‐like compounds, or other metabolites remain unknown. Metabolomic and targeted analytical approaches, including LC‐MS/MS, should be used to identify and quantify the components associated with antifungal and antibiofilm activity. Third, the study did not directly investigate mechanisms of action. Future work should evaluate hyphal transition, membrane permeability or integrity, extracellular‐matrix changes, and expression of genes involved in adhesion, filamentation, biofilm maturation, and stress responses. Fourth, all experiments were performed in vitro and therefore did not reproduce salivary flow, host immune responses, tissue interactions, or polymicrobial oral biofilms. These factors should be incorporated into more physiologically relevant models before efficacy is extrapolated to the oral cavity.

Fifth, cytotoxicity and biocompatibility were not assessed in oral epithelial cells, gingival fibroblasts, or other mammalian cell models; therefore, no conclusion regarding clinical safety can be drawn from the present data. Sixth, the study examined short‐term activity after 24‐h PM exposure and did not evaluate storage stability, persistence of antifungal activity over time, or sustained activity after incorporation into a clinically relevant formulation. Seventh, fixed concentrations were selected for the individual assays (2×MIC for mature‐biofilm disruption and 1/2 × MIC for the XTT assay), but a systematic concentration‐response analysis, exposure‐time optimization, and comparison of delivery approaches were not performed. Eighth, the PMs were not tested in combination with conventional antifungal agents; therefore, potential additive, synergistic, or antagonistic interactions remain unknown. These issues should be addressed in future dose‐optimization, formulation, stability, combination‐treatment, and safety studies.

Additional methodological limitations include the absence of a standard antifungal comparator (e.g., fluconazole) in the biofilm and metabolic‐activity assays, which limits direct benchmarking of PM efficacy. The pH of the PM preparations was adjusted to physiological pH (7.2–7.4), a step that may reduce the activity of pH‐sensitive organic acids such as lactic and acetic acid. Future studies should include uninoculated, pH‐adjusted MRS broth as a vehicle control and should evaluate PM activity across a defined pH range to clarify the contribution of organic acids. In addition, quantitative biofilm assays were performed in polystyrene 96‐well plates, whereas SEM analysis used tooth slabs, introducing a substrate variable that may affect biofilm architecture and limit direct cross‐method comparison. Standardization of the biofilm substrate across complementary assays would strengthen future studies.

## 5 Conclusions

The present in vitro findings demonstrate that LGG‐ and LR‐derived PMs suppress the metabolic activity of *C. albicans* biofilms, whereas a statistically significant reduction in viable biofilm counts was detected only with LGG PMs. SEM observations were consistent with altered biofilm architecture, particularly after LGG PM treatment. Under the conditions tested, LGG PMs showed a greater antibiofilm effect; however, these experiments do not establish clinical efficacy, safety, or a specific molecular mechanism.

Further research should evaluate diverse clinical and antifungal‐resistant *Candida* isolates; chemically characterize active metabolites using LC‐MS/MS or complementary metabolomic approaches; investigate hyphal transition, membrane integrity, biofilm‐associated gene expression, and other relevant mechanisms; and assess cytotoxicity in oral epithelial and fibroblast models. In vivo studies should subsequently evaluate efficacy and safety under conditions that incorporate salivary flow, host responses, tissue interactions, and polymicrobial communities. Dose‐response relationships, exposure time, formulation and delivery strategies, storage stability, sustained activity, and combinations with conventional antifungal agents should also be investigated before therapeutic or preventive applications are considered.

## Author Contributions

**Fatemeh Amirinia:** investigation, data curation, writing – original draft. **Morteza Banakar:** conceptualization, methodology, supervision, funding acquisition, writing – review and editing. **Mojgan Alaeddini:** conceptualization and methodology. **Shahroo Etemad‐Moghadam:** methodology, conceptualization, writing – review and editing. **Maryam Pourhajibagher:** investigation and formal analysis. **Maryam Noori:** investigation and data curation.

## Ethics Statement

This study was conducted with the approval and supervision of the National Institute for Medical Research Ethics Committee (IR.NIMAD.REC.1400.148). All procedures were carried out following the relevant guidelines and regulations.

## Consent

The authors have nothing to report.

## Conflicts of Interest

The authors declare no conflicts of interest.

## AI Tool Disclosure

In producing this manuscript, the authors used grammar and language assistance tools, including Grammarly and a large language model (Claude, Anthropic), to improve clarity and readability. Following use of these tools, all content was critically reviewed, revised as necessary, and approved by the authors, who take full responsibility for the accuracy, integrity, and originality of the published work.

## Data Availability

The data sets used and/or analyzed during the current study are available from the corresponding author upon reasonable request.
